# Timing of menarche and self-harm in adolescence and adulthood: a population-based cohort study

**DOI:** 10.1017/S0033291719002095

**Published:** 2020-09

**Authors:** Elystan Roberts, Abigail Fraser, David Gunnell, Carol Joinson, Becky Mars

**Affiliations:** 1 National Institute for Health Research Bristol Biomedical Research Centre, University Hospitals Bristol NHS Foundation Trust and University of Bristol, Bristol, UK; 2 Department of Population Health Sciences, University of Bristol, Bristol, UK; 3 Medical Research Council Integrated Epidemiology Unit at the University of Bristol, Bristol, UK

**Keywords:** ALSPAC, menarche, puberty, self-harm

## Abstract

**Background:**

Previous studies of pubertal timing and self-harm are limited by subjective measures of pubertal timing or by the conflation of self-harm with suicide attempts and ideation. The current study investigates the association between an objective measure of pubertal timing – age at menarche – and self-harm with and without suicidal intent in adolescence and adulthood in females.

**Method:**

Birth cohort study based on 4042 females from the Avon Longitudinal Study of Parents and Children (ALSPAC). Age at menarche was assessed prospectively between ages 8 and 17 years. Lifetime history of self-harm was self-reported at ages 16 and 21 years. Associations between age at menarche and self-harm, both with and without suicidal intent, were examined using multivariable logistic regression.

**Results:**

Later age at menarche was associated with a lower risk of lifetime self-harm at age 16 years (OR per-year increase in age at menarche 0.87; 95% CI 0.80–0.95). Compared with normative timing, early menarche (<11.5 years) was associated with an increased risk of self-harm (OR 1.31, 95% CI 1.04–1.64) and later menarche (>13.8 years) with a reduced risk (OR 0.74, 95% CI 0.58–0.93). The pattern of association was similar at age 21 years (OR per-year increase in age at menarche 0.92, 95% CI 0.85–1.00). There was no strong evidence for a difference in associations with suicidal *v.* non-suicidal self-harm.

**Conclusions:**

Risk of self-harm is higher in females with early menarche onset. Future research is needed to establish whether this association is causal and to identify potential mechanisms.

## Introduction

Self-harm is a major public health concern, affecting individuals both in clinical and community settings. There is evidence that rates of self-harm are rising, particularly among girls (Griffin *et al*., 2018). The incidence of self-harm peaks during adolescence (Gillies *et al*., 2018), which we define as ranging from age 10 years to age 19 years (World Health Organization, 1965, 1977), although the age range of adolescence has been the subject of debate (Sawyer *et al*., 2018). Recent population-based studies estimate lifetime risk of 18–19% in 12- to 17-year olds, with a considerably higher risk amongst females (Kidger *et al*., 2012; Geulayov *et al*., 2018).

Previous research has identified an association between an earlier timing of puberty relative to one's peers and an increased risk for a range of adverse outcomes in adolescence, including alcohol and substance misuse (Costello, 2007; Al-Sahab *et al*., 2012), conduct problems (Burt *et al*., 2006), eating disorders (Berger *et al*., 2009), depression (Stice *et al*., 2001) and depressive symptoms (Rierdan and Koff, 1991; Hayward *et al*., 1999; Joinson *et al*., 2013). Few studies investigate the persistence of associations into adulthood. In those that do, the associations appear to attenuate in later adolescence and early adulthood (Joinson *et al*., 2013; Senia *et al*., 2018), though there is some evidence for persistent effects (Stattin and Magnusson, 1990; Copeland *et al*., 2010; Lien *et al*., 2010).

Earlier timing of puberty has been associated with an increased risk of suicide attempts, in both cross-sectional (Graber *et al*., 1997; Larsson and Sund, 2008; Tondo *et al*., 2017) and prospective designs (Wichstrøm, 2000). In a sample of 12- to 15-year-old US and Australian adolescents, Patton *et al*. (2007) reported a cross-sectional association between later self-reported pubertal stage, measured using the Pubertal Development Scale (PDS; Petersen *et al*., 1988), and adolescent self-harm. The authors concluded that adolescents who started puberty earlier, and so were in later pubertal stages at the time of measurement, were at higher risk of self-harm than their peers. Cross-sectional studies in Chinese adolescents have also found evidence that earlier developing females (indexed via age at menarche) were at greater risk of self-harm (Deng *et al*., 2011; Chen *et al*., 2017). However, findings have been mixed and a number of studies have not found evidence for an effect (Riesch *et al*., 2008; Chiang *et al*., 2010; Liu *et al*., 2018; Jung *et al*., 2019).

Some of this inconsistency may have arisen from the use of subjective measures of pubertal timing. For example, a modified, single-item version of the Pubertal Development Scale, which has been used in a number of studies (Wichstrøm, 2000; Larsson and Sund, 2008) asks participants how they perceived the progress of their pubertal development compared to their peers. This item could have been problematic in relying not only on participants' perceptions of their development, but also the development of their peers. Responses could have been affected by requiring respondents to make social judgments in reporting self-perceptions. Indeed, assessments of the validity of pubertal timing measures have concluded that perceived relative timing is biased towards the average compared to measures which do not require social comparison (Alsaker, 1992).

Of those studies which have examined the association between pubertal timing and self-harm, few have distinguished between self-harm with and without suicidal intent. This distinction may be important, as there has been debate as to whether suicidal and non-suicidal self-harm (NSSH) are part of the same continuum (Kapur *et al*., 2013) or whether they are distinct behaviours (Muehlenkamp and Kerr, 2010). Previous longitudinal research found that suicidal and non-suicidal self-harm had both unique and common risk factors, and concluded that they were overlapping behaviours, but with some distinct characteristics (Chang *et al*., 2014; Mars *et al*., 2014*a*). In addition, no studies have prospectively investigated whether associations between pubertal timing and self-harm persist into adulthood.

The current study uses data from a large UK birth cohort to examine the association between age at menarche and self-harm. We address limitations of earlier studies by using a prospective design, including an objective measure of pubertal timing (age at menarche), and adjusting for a range of confounders. Our primary aims are to assess whether there is an association between earlier timing of menarche and self-harm at age 16 years, and whether this association persists into early adulthood (21 years). Our secondary aim is to examine whether the association differs for NSSH and self-harm with suicidal intent.

## Methods

### Participants

The sample was drawn from the Avon Longitudinal Study of Parents and Children (ALSPAC), an ongoing population-based cohort study. Pregnant women who had an estimated date of delivery between 1 April 1991 and 31 December 1992, and who resided in the former county of Avon in the south-west of England, were recruited (Boyd *et al*., 2013; Fraser *et al*., 2013). Both parents and children in the study have been followed up regularly since recruitment through questionnaires and research clinic visits. Ethical approval for the study was obtained from the ALSPAC Law and Ethics committee and local research ethics committees, and informed consent for the use of data collected via questionnaires and clinics was obtained from participants following the recommendations of the ALSPAC Ethics and Law Committee at the time. The ALSPAC study website contains more information, including details of all the data that is available through a fully searchable data dictionary (http://www.bristol.ac.uk/alspac/researchers/our-data/). From an initial core sample of singletons or first-born twins alive at 1 year of age (*N* = 13 793), of whom *n* = 6676 were female, those with data available on age at menarche were eligible for inclusion in the study (*n* = 4042). Of those females who provided age at menarche data, 1282 (31.66%) also had complete data on self-harm and all confounders (online Supplementary Fig. S1).

### Measures

#### Age at menarche

Participants completed up to nine annual postal questionnaires relating to pubertal development from the age of 8–17 years. Each questionnaire asked whether menstruation had started, and if so at what age (in years and months). Earlier questionnaires were typically completed by the study mothers on their daughter's behalf, but the proportion of questionnaires completed by the study children increased with age. The first-reported age at menarche was used. The continuous measure of age at menarche was categorised based on the distribution of the variable into normative (11.5–13.8 years: mean age at menarche ±1 standard deviation) as well as early (<11.5 years) and late (>13.8 years) menarche, in line with previous definitions of menarcheal timing (Stice *et al*., 2001; Joinson *et al*., 2013).

#### Lifetime, suicidal and non-suicidal self-harm

Participants received postal questionnaires at age 16 and 21 years which included questions on self-harm. These questions were based on those used in the Child and Adolescent Self-harm in Europe (CASE) study (Madge *et al*., 2008). Participants answered the question ‘Have you ever hurt yourself on purpose in any way (e.g. by taking an overdose of pills or by cutting yourself)?’. Those who responded positively were classified as having a history of self-harm. Participants were classified as having a history of self-harm by age 21 years if they responded positively at either age 16 or 21 years. Suicidal intent was established at age 16 years from two follow-up questions. Participants were classified as having self-harmed with suicidal intent if they responded positively to the question ‘On any of the occasions when you have hurt yourself on purpose, have you ever seriously wanted to kill yourself?’, or if they selected the option ‘I wanted to die’ when responding to the question ‘Do any of the following reasons help to explain why you hurt yourself on that [the most recent] occasion?’.

#### Confounders

We considered the following as potential confounding factors based on previous literature: socioeconomic status as measured by maternal education level (lower than O-levels, O-levels, A-levels, degree; O-levels and A-levels are British school examinations taken at around age 16 and 18 years, respectively) and material hardship [assessed at age 5 years by asking mothers ‘How difficult at the moment do you find it to afford these items? Food, clothing, heating, rent, items for child’. Participants scored each item from 1 (very difficult) to 4 (not difficult). A total material hardship score was calculated by taking a participants' total score from these five variables and subtracting it from 20, to provide a score range from 0 (lowest level of hardship) to 15 (highest level of hardship); Joinson *et al*., 2017]; maternal depression dichotomised at a cut-off score of 12 on the Edinburgh Postnatal Depression Scale (EPDS; Cox *et al*., 1987), collected during pregnancy; childhood sexual abuse retrospectively self-reported at age 22 (Magnus *et al*., 2018); parental separation reported by mothers before the child's fifth birthday (Culpin *et al*., 2015); and body mass index (BMI) at age 9, calculated based on weight and height measured at research clinics or from self-reported height and weight where clinic data were missing.

### Statistical analysis

All analyses were conducted using Stata version 15 (Stata Inc., 2017). Associations were examined between age at menarche (both the continuous and categorical variables) and self-harm at age 16 and age 21 years using logistic regression analyses. Secondary analyses used multinomial logistic regression to investigate the associations between age at menarche and self-harm with and without suicidal intent. Both unadjusted analyses and analyses controlling for confounding factors were conducted.

### Missing data

Complete data on exposure, outcomes and confounders were available for 1279 participants. Primary analyses were conducted on imputed datasets that included individuals with complete age at menarche data (*n* = 4042). Of those individuals with complete exposure data, 2706 (67.0%) had complete outcome data at age 16 years, and 2272 (56.2%) had complete outcome data at age 21 years. Missing outcome and confounder data were imputed using Multivariable Imputation by Chained Equations (Royston and White, 2011). The use of this method is based on the Missing at Random (MAR) assumption that conditional on the variables included in the imputation model, there are no systematic differences between observed and missing values for a given variable (Sterne *et al*., 2009). Fifty imputed datasets were produced. The imputation models included all variables used in the analysis as well as relevant auxiliary variables (see online Supplement). These included measures of socioeconomic status, demographic data, mental health and substance use data, and earlier or later recordings of variables of interest such as maternal depression. Monte Carlo errors are available on request.

## Results

Table 1 shows the distributions of observed and imputed data. The final distributions were similar in both datasets. Females who provided age at menarche data constituted the observed complete case sample. These participants were more likely to be white, to have a more educated mother in a higher social class, and to have lived with both parents until age 5 compared to those who did not provide data on age at menarche (online Supplementary Table S2).

**Table 1.** Distributions of values of exposure, outcome, and confounder variables observed in participants with complete data for all included variables, and distributions in imputed datasets

			Distribution *n* (%) for categorical variables Mean (s.e.) for continuous variables	
Imputed variable		*n* (%) data missing	Observed data (*n* = 1282)	Imputed datasets (*n* = 4042)
Timing of menarche	Early	0	166 (12.95)	15.91
	Normative	0	880 (68.64)	67.64
	Late	0	236 (18.41)	16.45
Self-harm (age 16 years)		1342 (33.14)	302 (23.56)	25.26
Non-suicidal self-harm (age 16 years)			212 (16.54)	17.41
Self-harm with suicidal intent (age 16 years)			90 (7.02)	7.85
Self-harm (age 21 years)		1165 (28.77)	384 (32.08)	34.45
Maternal education	<O-level	168 (4.15)	180 (14.04)	24.20
	O-level		422 (32.92)	34.75
	A-level		377 (29.41)	25.34
	Degree		303 (23.63)	15.71
Maternal depression		521 (12.87)	99 (7.72)	11.53
Sexual abuse		1895 (46.80)	64 (4.99)	6.25
Parental separation		0	116 (9.05)	15.17
Material hardship		949 (23.44)	1.60 (2.55)	2.07 (0.51)
Body mass index (BMI)		628 (15.51)	17.75 (2.79)	17.98 (0.49)

Proportions are displayed for imputed datasets.

A quarter (25.3%) of respondents reported having ever self-harmed at age 16 years. This rose to 34.5% by the age of 21 years. Of the individuals who had self-harmed at age 16 years, 31.1% reported having done so with suicidal intent. Within each category of pubertal timing, the proportion of participants reporting self-harm at age 16 years was highest in those who experienced early menarche (31.8%), and lowest in those who experienced late menarche (19.4%) with 25.1% reporting self-harm in the normative timing of menarche category (Table 2).

**Table 2.** Distribution of outcome and confounder variables in each category of timing of menarche in imputed data

		Distribution Proportion (s.e.) for categorical variables Mean (s.e.) for continuous variables		
Variable		Early (<11.5 years)	Normative (11.5–13.8 years)	Late (>13.8 years)
Self-harm (age 16 years)		31.81 (2.26)	25.14 (1.00)	19.43 (1.71)
Non-suicidal self-harm (age 16 years)		21.14 (1.97)	17.61 (0.86)	13.00 (1.46)
Self-harm with suicidal intent (age 16 years)		10.67 (1.46)	7.53 (0.64)	6.43 (1.08)
Self-harm (age 21 years)		40.50 (2.74)	34.05 (1.40)	30.23 (2.18)
Maternal education	<O-level	27.60 (1.81)	24.43 (0.84)	20.00 (1.59)
	O-level	35.98 (1.94)	34.90 (0.94)	32.94 (1.86)
	A-level	23.19 (1.71)	24.78 (0.84)	29.71 (1.80)
	Degree	13.24 (1.37)	15.89 (0.71)	17.34 (1.48)
Maternal depression		15.00 (1.51)	10.87 (0.63)	10.90 (1.25)
Sexual abuse		7.96 (1.53)	6.19 (0.63)	4.84 (1.14)
Parental separation		18.35 (1.53)	14.59 (0.68)	14.44 (1.36)
Material hardship		2.32 (0.14)	2.06 (0.06)	1.85 (0.12)
Body mass index (BMI)		19.38 (0.13)	17.98 (0.06)	16.63 (0.10)

### Lifetime self-harm at age 16 years

Unadjusted and adjusted odds ratios for the association between age at menarche and lifetime self-harm at age 16 years are presented in Table 3. Later age at menarche was associated with a reduced risk of self-harm in adolescence. This association remained after adjustment for confounders (per-year increase in age at menarche OR 0.87, 95% CI 0.80–0.95). Compared with the normative reference group those with early menarche had an increased risk of self-harm, whereas those with later menarche had a decreased risk. Results remained after adjustment for confounders (early menarche OR 1.31, 95% CI 1.04–1.64; late menarche OR 0.74, 95% CI 0.58–0.93).

**Table 3.** Odds ratios showing associations between age at menarche and self-harm at age 16 and age 21

	Age 16				Age 21			
	Unadjusted OR (95% CI)	*p*	Adjusted OR (95% CI)	*p*	Unadjusted OR (95% CI)	*p*	Adjusted OR (95% CI)	*p*
Per 1-year later age at menarche	0.85 (0.78–0.92)	<0.001	0.87 (0.80–0.95)	0.001	0.89 (0.82–0.96)	0.004	0.92 (0.85–1.00)	0.062
Timing of menarche								
Early (<11.5 years)	1.39 (1.11–1.74)	0.004	1.31 (1.04–1.64)	0.022	1.32 (1.05–1.65)	0.018	1.22 (0.96–1.54)	0.104
Normative (11.5–13.8 years)	1.00	–	1.00	–	1.00	–	1.00	–
Late (>13.8 years)	0.72 (0.57–0.91)	0.006	0.74 (0.58–0.93)	0.012	0.84 (0.68–1.04)	0.103	0.88 (0.71–1.09)	0.240

Analyses completed on imputed datasets (*n* = 4042).

Adjusted models include measures of maternal education, material hardship, maternal depression, childhood sexual abuse, parental separation, and body mass index (BMI).

### Lifetime self-harm at age 21 years

Odds ratios for the association between age at menarche and lifetime self-harm by age 21 years are also presented in Table 3. Findings are consistent with the age 16 years analyses, showing an association between later age at menarche and a reduced risk of self-harm when age at menarche was assessed continuously (OR per-year increase in age at menarche 0.92; 95% CI 0.85–1.00). The same pattern of results was also found when menarche was defined categorically, however the confidence intervals included the null.

### Suicidal *v.* non-suicidal *v.* no self-harm

Results of the multinomial logistic regression analyses examining the association between age at menarche and self-harm with and without suicidal intent are presented in Table 4. The comparison group in these analyses was adolescents who had never self-harmed. The results suggest that a 1-year increase in age at menarche was associated with a lower risk of both NSSH (RRR 0.86; 95% CI 0.78–0.94) and self-harm with suicidal intent (RRR 0.90; 95% CI 0.79–1.02). When timing of menarche was examined categorically, there was weak evidence for an association between early menarche and both NSSH (RRR 1.26; 95% CI 0.97–1.64) and suicidal self-harm (RRR 1.42; 95% CI 0.99–2.02) compared to the normative reference group, however an association with late menarche was found only for NSSH (RRR 0.68; 95% CI 0.52–0.92). To compare whether associations differed for self-harm with and without suicidal intent, we estimated the model with an alternative reference group (those with NSSH; Table 4). These analyses did not provide any strong evidence for a difference in the association for NSSH and self-harm with suicidal intent. Similar findings were also observed by age 21 years (online Supplementary Table S6).

**Table 4.** Relative risk ratios showing associations between age at menarche and suicidal and non-suicidal self-harm, *v.* no self-harm, and suicidal *v.* non-suicidal self-harm, at age 16

	Non-suicidal self-harm *v*. no self-harm				Suicidal self-harm *v*. no self-harm				Suicidal *v*. non-suicidal self-harm			
	Unadjusted		Adjusted		Unadjusted		Adjusted		Unadjusted		Adjusted	
	RRR (95% CI)	*p*	RRR (95% CI)	*p*	RRR (95% CI)	*p*	RRR (95% CI)	*p*	RRR (95% CI)	p	RRR (95% CI)	*p*
Per 1-year increase in aPHV	0.85 (0.77–0.92)	<0.001	0.86 (0.78–0.94)	0.013	0.86 (0.76–0.97)	0.002	0.90 (0.79–1.02)	0.087	1.02 (0.89–1.16)	0.823	1.04 (0.91–1.20)	0.540
Timing of aPHV												
Early (<11.0 years)	1.31 (1.02–1.71)	0.037	1.26 (0.97–1.64)	0.089	1.55 (1.09–2.20)	0.014	1.42 (0.99–2.02)	0.056	1.18 (0.79–1.76)	0.419	1.13 (0.75–1.69)	0.570
Normative (11.0–12.6 years)	1.00	–	1.00	–	1.00	–	1.00	–	1.00	–	1.00	–
Late (>12.6 years)	0.69 (0.52–0.90)	0.007	0.69 (0.52–0.92)	0.011	0.79 (0.53–1.18)	0.248	0.84 (0.56–1.25)	.379	1.15 (0.73–1.83)	0.541	1.20 (0.75–1.93)	0.441

Analyses completed on imputed datasets (*n* = 4042).

Adjusted models include measures of maternal education, material hardship, maternal depression, childhood sexual abuse, parental separation, and body mass index (BMI).

### Comparison between complete case and imputed data

Comparison between the complete case and imputed data is shown in online Supplementary Tables S3 and S4b. Overall the pattern of results was consistent, however there was no association between early menarche and self-harm found in the complete case sample.

## Discussion

### Main findings

We found strong evidence of an inverse association between age at menarche and self-harm in adolescence: odds of self-harm decreased by 13% (95% CI 5–20%) for every 1-year increase in age at menarche. Findings were consistent with a linear association in the imputed dataset. The results were consistent, although attenuated slightly, at age 21 years.

We did not find evidence for a differential effect of age at menarche on self-harm with and without suicidal intent. This is consistent with previous studies investigating the association between pubertal timing and self-harm (Larsson and Sund, 2008; Deng *et al*., 2011).

### Strengths and limitations

A notable strength of the current study is the use of a large, population-based cohort sample. This is important as most young people who self-harm do not present to specialist services (Hawton *et al*., 2002; Kidger *et al*., 2012). Data on age at menarche were collected regularly throughout childhood and adolescence, minimising the risk of recall error. Age at menarche is an objective and salient measure of pubertal timing (Dorn and Biro, 2011). The availability of self-harm data from two separate time points enabled us to investigate whether the effect of age at menarche on self-harm risk persists into early adulthood. We were also able to adjust for a wide range of confounding variables, though some residual confounding may still be possible.

Our findings need to be interpreted in light of some limitations. First, as for most cohort studies, there has been a loss to follow-up in ALSPAC, which may have biased our complete case analysis. However, missing data were imputed up to the full sample of individuals with data on timing of menarche (*n* = 4042). Multiple imputation relies on the MAR assumption that there are no systematic differences between observed and missing values for a variable given all the variables in the imputation model. The large pool of variables available in the ALSPAC dataset allowed us to include a high number of relevant auxiliary variables, maximising confidence in the MAR assumption.

A second limitation is the possibility for measurement error in the self-harm variable, which was assessed via self-report (Mars *et al*., 2016). When deriving lifetime self-harm at age 21 years, we included participants who had responded positively to a self-harm question at either age time point. However, for some participants reports were not consistent across time. Nevertheless, findings of the age 21 years analysis were consistent when only individuals who reported self-harm at age 21 years were included (online Supplementary Table S5). It can also be difficult to reliably establish suicidal intent: 23% (*n* = 40) of individuals who said that they ‘wanted to die’ when they most recently self-harmed responded negatively to the question of whether they had ever ‘seriously wanted to kill [themselves]’. As with previous studies (Nock, 2010), we classified participants as having attempted suicide if they reported any – non-zero-level of suicidal intent.

The use of a lifetime self-harm variable means that reported self-harm could have preceded menarche for some individuals. This could have led to reverse causality, as there is some evidence that psychological distress may lead to an earlier age at menarche (Mishra *et al*., 2009). However, the incidence of self-harm is much less common in childhood than in adolescence (Whitlock and Selekman, 2014). The current study examined the association between age at menarche and lifetime history of self-harm at two time points: during adolescence and early adulthood. However, previous research (Moran *et al*., 2012; Mars *et al*., 2014*b*) has suggested that patterns of self-harm behaviour may fluctuate between these two periods. We were also unable to distinguish individuals who only self-harmed with suicidal intent from those who had engaged in both suicidal and non-suicidal self-harm.

### Relevance to wider literature

The prevalence of self-harm observed in our results is in line with previous studies of adolescent females (Brunner *et al*., 2014; Morey *et al*., 2016). Our results support the findings of previous studies on pubertal timing and self-harm, which have generally found that earlier timing of puberty is associated with greater risk of self-harm and suicidal behaviour (Graber *et al*., 1997; Wichstrøm, 2000; Patton *et al*., 2007; Larsson and Sund, 2008; Deng *et al*., 2011; Chen *et al*., 2017; Tondo *et al*., 2017). The measure of pubertal timing used in this study relies less on social comparison than measures in some previous studies, which have used more subjective assessment measures. For example, Larsson and Sund (2008) found differences in the proportion of individuals perceiving their development as ‘Much earlier than my peers’ between those who reported self-harm and those who did not, and Wichstrøm (2000) showed a positive association between self-harm and perceived pubertal timing (measured from 1 – ‘Much later’ to 7 – ‘Much earlier’). Further, many studies have not analysed pubertal timing and self-harm as unique exposure and outcome variables: Patton *et al*. (2007), for example, measured pubertal stage, not pubertal timing, and Tondo *et al*. (2017) used a suicidality measure which combined suicidal ideation and attempts, but did not examine self-harm alone. The current study is the first to explicitly measure age at menarche and self-harm in a European population.

Early timing of menarche has been associated with depression (Stice *et al*., 2001) and depressive symptoms (Joinson *et al*., 2013; Sequeira *et al*., 2017). Given depression is one of the strongest risk factors for self-harm (Hawton *et al*., 2012; Kidger *et al*., 2012), it may mediate the association between age at menarche and self-harm. Alternatively, both associations may share similar underlying mechanisms. For example, Angold *et al*. (1999) showed in a multivariable model that the association between pubertal stage and depression was explained by measures of testosterone and oestradiol. These same hormonal factors may be involved in the association between puberty and self-harm.

It has been suggested that the hormones involved in pubertal development may have a direct influence on neural structures such as the hippocampus and the amygdala via dopaminergic and serotonergic pathways, and that this has an effect on depressive symptoms (Angold and Costello, 2006). Indeed, there is evidence of substantial neurological change in these areas during puberty (Goddings *et al*., 2014). The disparity in the development of reward-seeking pathways *v.* the executive function-associated prefrontal cortex has been posited as a possible explanation for the increase in risky behaviours like self-harm observed in adolescents, with early developers at particularly increased risk (Steinberg, 2008; Somerville *et al*., 2010).

An alternative explanation for the findings lies in the psychosocial development involved in puberty. Early developers may find themselves very physically different from their peers, and comparison with peers may lead to psychosocial stress which could then lead to self-harm. Our results fit within the early timing hypothesis (Stattin and Magnusson, 1990; Caspi and Moffitt, 1991), which proposes that early maturing females experience the greatest adverse psychological effects of puberty because they are not yet cognitively equipped for the psychosocial pressures which accompany it. Indeed, early-developing girls are more likely to be perceived as older than they are (Sontag *et al*., 2011) and to engage in sexual relationships earlier (Copeland *et al*., 2010). They may be treated with expectations that they are emotionally or cognitively unable to meet, or may experience earlier exposure to stressors like relationship problems, which are a risk factor for self-harm (Silva *et al*., 2017). Romantic relationship stress has been associated with an increased risk of non-suicidal self-injury in early-developing girls (Miller *et al*., 2018). Conversely, late-developing girls may benefit from exposure to early- and normatively-timed developers experiencing the effects of puberty before they experience it themselves.

In using timing of menarche as our measure for pubertal timing it was necessary to exclude males from our sample. The findings of studies which have reported the associations of pubertal timing with self-harm in boys have been inconsistent. For example, Patton *et al*. (2007) found no evidence that associations between pubertal timing and self-harm differed in males and females, whereas Wichstrøm (2000) identified a quadratic relationship between pubertal timing and suicide attempts in boys, where both early- and late-developing boys were at increased risk. Future research should aim to clarify the association between pubertal timing and self-harm in males.

In addition, future research should aim to address the ongoing question of the effect of pubertal timing *v.* pubertal stage. The current study examined self-harm at the same chronological time points for all participants, but future research should consider examining self-harm at the same pubertal time point (e.g. 6 months post-menarche) for all participants. This would help to clarify whether it is becoming pubertal or the timing of becoming pubertal that is associated with self-harm risk.

Further research is also needed to examine whether the association between timing of menarche and self-harm identified in the current research is causal. It would also be valuable to identify modifiable mechanisms such as depression, neurocognitive development, and psychosocial functioning that may explain our findings. Identifying these factors will enable the development of interventions which can be targeted at early developing girls to reduce their risk of self-harm.

## Conclusions

Pubertal timing is inversely associated with self-harm in females, with older age at menarche associated with a reduced risk of lifetime self-harm both in adolescence and in early adulthood. These results add support to the theory that early pubertal timing is a risk factor for mental health problems in adolescent and young adult females. Early-developing females should be a focus on targeted mental health intervention.

## Supplementary material

For supplementary material accompanying this paper visit https://doi.org/10.1017/S0033291719002095. click here to view supplementary material
