# Supplementary material for: Timing of menarche and self-harm in adolescence and adulthood: a population-based cohort study
Source: Psychol Med. 2019 Aug 28;50(12):2010–8. doi: 10.1017/S0033291719002095 (PMC7525770; doi:10.1017/S0033291719002095)
Supplement: Supplementary file 1 [file S0033291719002095sup001.docx]

Table S1 – Multiple Imputation Model

All participants with data on age at menarche were included in analyses. Outcome and covariate data were imputed. The imputation model included all variables used in the analysis (exposures, outcomes, and confounders), in addition to auxiliary variables listed below. Bespoke combinations of auxiliary data, specific to each imputation model, were utilised.

| Variable name | Details | Type of variable |
| --- | --- | --- |
| **Demographic variables** |  |  |
| Crowding | Number of individuals in the household >4; collected at 8 weeks’ gestation | Categorical |
| Parity | Number of siblings of study child; collected at 18 weeks’ gestation | Continuous |
| Social class | Categorised based on parents’ combined level of social class on the Registrar General’s scale; collected at 32 weeks’ gestation | Ordered categorical (4 categories) |
| Child IQ | Based on the Wechsler Intelligence Scale for Children (WISC); collected at 8 years at a research clinic | Continuous |
| Family adversity | Including measures of early parenthood, housing adequacy, and partner cruelty; collected up to child age 4 years at research clinics and using questionnaires | Categorical |
| **Family mental health** |  |  |
| Maternal suicide attempt | Collected up until age 9 years using questionnaires | Categorical |
| Maternal depression | Collected at child age 8 weeks and 21 years old using the Edinburgh Postnatal Depression Scale | Continuous |
| Exposure to family self-harm | Child-reported, collected at age 16 using a questionnaire | Categorical |
| Exposure to maternal self-harm | Child-reported, collected at age 16 using a questionnaire | Categorical |
| **Parent substance use** |  |  |
| Maternal cannabis use | Mother-reported, collected at child age 7 and 9 years | Categorical |
| **Child mental health** |  |  |
| Child depressive disorder | Collected at age 15 years using the Development and Wellbeing Assessment (DAWBA) scale and at age 18 years using the Clinical Interview Schedule – Revised (CISR) | Categorical |
| Child anxiety disorder | Collected at age 15 years using the Development and Wellbeing Assessment (DAWBA) scale and at age 18 years using the Clinical Interview Schedule – Revised (CISR) | Categorical |
| Child depressive symptoms | Collected at age 10, 12, 13, 16, 17, and 18 years using the Mood and Feelings Questionnaire (MFQ) | Categorical |
| **Child substance use** |  |  |
| Child smoking | Collected at age 13, 16, and 17 years at research clinics and at age 14 years using a questionnaire | Categorical |
| Child heavy alcohol use | Collected at age 13 and 16 years at research clinics | Categorical |
| Child cannabis use | Collected at age 13, 16, and 17 years at research clinics | Categorical |
| Child illicit drug use | Collected at age 16 years at a research clinic and using a questionnaire | Categorical |
| **Previous self-harm** |  |  |
| Lifetime self-harm | Collected at age 11, 15, and 18 years at research clinics | Categorical |

Table S2 – Observed values of descriptive data in females who did and did not provide data on age at menarche in the core ALSPAC sample. Total *n* is displayed in column headings; numbers of participants for each covariate vary based on amount of missing data.

| Variable | Description | N | Data on age at menarche  (n = 4,042) | No data on age at menarche  (n = 2,634) | X^2^ | P |
| --- | --- | --- | --- | --- | --- | --- |
| Self-harm at age 16 | Yes | 726 | 688 / 2,706 (25.42%) | 38 / 131  (29.01%) | 0.84 | .359 |
|  | No | 2,111 | 2,018 / 2,706 (74.58%) | 93 / 131  (70.99%) |  |  |
| Maternal education level | < O level | 1,760 | 913 / 3,874 (23.57%) | 847 / 2,063 (41.06%) | 249.99 | <.001 |
|  | O level | 2,051 | 1,349 / 3,874 (34.82%) | 702 / 2,063 (34.03%) |  |  |
|  | A level | 1,344 | 993 / 3,874  (25.63%) | 351 / 2,063 (17.01%) |  |  |
|  | Degree or higher | 782 | 619 / 3,874  (15.98%) | 163 / 2,063  (7.9%) |  |  |
| Material hardship category | <5 | 3,434 | 2,783 / 3,093  (89.98%) | 651 / 754 (86.34%) | 10.69 | .005 |
|  | 6-10 | 329 | 242 / 3,093  (7.82%) | 87 / 754  (11.54%) |  |  |
|  | 11-15 | 84 | 68 / 3,093  (2.20%) | 16 / 754  (2.12%) |  |  |
| Highest parental social class | Professional/managerial | 3,040 | 2,192 / 3,684  (59.50%) | 848 / 1,842  (46.04%) | 89.94 | <.001 |
|  | Other | 2,486 | 1,492 / 3,684  (40.50%) | 994 / 1,842  (53.96%) |  |  |
| Parental separation before child’s 5^th^ birthday | Yes | 1,092 | 613 / 4,042  (15.17%) | 479 / 2,634  (18.19%) | 10.63 | .001 |
|  | No | 5,584 | 3,429 / 4,042  (84.83%) | 2,155 / 2,634  (81.81%) |  |  |
| Child’s ethnicity | White | 5,747 | 3,789 / 3,866  (98.01%) | 1,958 / 2,030 (96.45%) | 13.07 | <.001 |
|  | Other | 149 | 77 / 3,866  (1.99%) | 72 / 2,030  (3.55%) |  |  |

Table S3 Odds ratios showing associations between age at menarche and self-harm at age 16 and 21 years in the complete case sample (n = 1,282) and in the imputed datasets (n = 4,042). All models adjusted for measures of maternal education, material hardship, maternal depression, childhood sexual abuse, parental separation, and body mass index (BMI).

|  | Age 16 | | | | Age 21 | | | |
| --- | --- | --- | --- | --- | --- | --- | --- | --- |
|  | Complete case OR (95% CI) | p | Imputed datasets OR (95% CI) | p | Complete case OR (95% CI) | p | Imputed datasets OR (95% CI) | p |
| Per one-year later age at menarche | 0.90  (0.80 – 1.01) | .083 | 0.87  (0.80 - 0.95) | .001 | 0.93  (0.83 – 1.05) | .226 | 0.92  (0.85 – 1.00) | .062 |
| Timing of menarche | | | | | | | | |
| *Early*  *(<11.5 years)* | 0.91  (0.61 – 1.35) | .641 | 1.31  (1.04 – 1.64) | .022 | 1.01  (0.70 – 1.46) | .964 | 1.22  (0.96 – 1.54) | .104 |
| *Normative*  *(11.5-13.8 years)* | 1.00 | - | 1.00 | - | 1.00 | - | 1.00 | - |
| *Late*  *(>13.8 years)* | 0.57  (0.39 – 0.84) | .005 | 0.74  (0.58 - 0.93) | .012 | 0.72  (0.51 – 1.03) | .069 | 0.88  (0.71 – 1.09) | .240 |

Table S4a Relative risk ratios showing associations between age at menarche and suicidal and non-suicidal self-harm, versus no self-harm, at age 16 years in the complete case sample (n = 1,282) and in the imputed datasets (n = 4,042). All models adjusted for measures of maternal education, material hardship, maternal depression, childhood sexual abuse, parental separation, and body mass index (BMI).

|  | Non-suicidal self-harm v no self-harm | | | | Suicidal self-harm v no self-harm | | | |
| --- | --- | --- | --- | --- | --- | --- | --- | --- |
|  | Complete case RRR (95% CI) | p | Imputed datasets RRR (95% CI) | p | Complete case RRR (95% CI) | p | Imputed datasets RRR (95% CI) | p |
| Per one-year later age at menarche | 0.87  (0.76 – 1.00) | .048 | 0.86  (0.78 - 0.94) | .013 | 0.97  (0.79 – 1.18) | .745 | 0.90  (0.79 – 1.02) | .087 |
| Timing of menarche | | | | | | | | |
| *Early*  *(<11.5 years)* | 0.83  (0.52 – 1.32) | .419 | 1.26  (0.97 – 1.64) | .089 | 1.12  (0.61 – 2.06) | .704 | 1.42  (0.99 – 2.02) | .056 |
| *Normative*  *(11.5-13.8 years)* | - | - | - | - | - | - | - | - |
| *Late*  *(>13.8 years)* | 0.49  (0.31 – 0.79) | .003 | 0.69  (0.52 - 0.92) | .011 | 0.78  (0.41 – 1.48) | .451 | 0.84  (0.56 – 1.25) | .379 |

Table S4b Relative risk ratios showing associations between age at menarche and suicidal versus non-suicidal self-harm at age 16 years in the complete case sample (n = 1,282) and in the imputed datasets (n = 4,042). All models adjusted for measures of maternal education, material hardship, maternal depression, childhood sexual abuse, parental separation, and body mass index (BMI). Model re-estimated using non-suicidal self-harm as the reference group.

|  | Suicidal v non-suicidal self-harm | | | |
| --- | --- | --- | --- | --- |
|  | Complete case RRR (95% CI) | p | Imputed datasets RRR (95% CI) | p |
| Per one-year later age at menarche | 1.11  (0.88 – 1.40) | .360 | 1.04  (0.91 – 1.20) | .540 |
| Timing of menarche |  |  |  |  |
| *Early*  *(<11.5 years)* | 1.36  (0.67 – 2.77) | .393 | 1.13  (0.75 – 1.69) | .570 |
| *Normative*  *(11.5-13.8 years)* | 1.00 | - | 1.00 | - |
| *Late*  *(>13.8 years)* | 1.58  (0.75 – 3.35) | .229 | 1.20  (0.75 – 1.93) | .441 |

Table S5 Odds ratios showing associations between age at menarche and self-harm reported at age 21 years.

Footnote: Analyses completed on imputed datasets (n=4,042).

Adjusted models include measures of maternal education, material hardship, maternal depression, childhood sexual abuse, parental separation, and body mass index (BMI).

|  | Age 21 | | | | |
| --- | --- | --- | --- | --- | --- |
|  | | Unadjusted OR (95% CI) | p | Adjusted OR (95% CI) | p |
| Per one-year later age at menarche | | 0.91  (0.84 – 0.98) | .016 | 0.94  (0.87 – 1.03) | .183 |
| Timing of menarche | |  |  |  |  |
| *Early*  *(<11.5 years)* | | 1.25  (0.99 – 1.58) | .061 | 1.15  (0.90 – 1.47) | .257 |
| *Normative*  *(11.5-13.8 years)* | | 1.00 | - | 1.00 | - |
| *Late*  *(>13.8 years)* | | 0.85  (0.68 – 1.07) | .167 | 0.90  (0.71 – 1.13) | .359 |

Table S6 Relative risk ratios showing associations between age at menarche and suicidal and non-suicidal self-harm, versus no self-harm, and suicidal versus non-suicidal self-harm, at age 21 years.

Footnote: Analyses completed on imputed datasets (n=4,042).

Adjusted models include measures of maternal education, material hardship, maternal depression, childhood sexual abuse, parental separation, and body mass index (BMI).

|  | **Non-suicidal self-harm v no self-harm** | | | | **Suicidal self-harm v no self-harm** | | | | **Suicidal v non-suicidal self-harm** | | | |
| --- | --- | --- | --- | --- | --- | --- | --- | --- | --- | --- | --- | --- |
|  | Unadjusted | | Adjusted | | Unadjusted | | Adjusted | | Unadjusted | | Adjusted | |
|  | RRR (95% CI) | p | RRR (95% CI) | p | RRR (95% CI) | p | RRR (95% CI) | p | RRR (95% CI) | p | RRR (95% CI) | p |
| Per one-year later age at menarche | 0.94  (0.87 – 1.03) | .171 | 0.97  (0.88 – 1.06) | .452 | 0.85  (0.75 – 0.97) | .013 | 0.91  (0.79 – 1.04) | .158 | 0.90  (0.78 – 1.04) | .163 | 0.94  (0.80 – 1.10) | .422 |
| Timing of menarche | | | | | | | | | | | | |
| *Early*  *(<11.5 years)* | 1.14  (0.88 – 1.48) | .309 | 1.08  (0.83 – 1.42) | .557 | 1.44  (1.01 – 2.06) | .045 | 1.27  (0.88 – 1.83) | .209 | 1.26  (0.83 – 1.91) | .273 | 1.17  (0.76 – 1.79) | .474 |
| *Normative*  *(11.5-13.8 years)* | 1.00 | - | 1.00 | - | 1.00 | - | 1.00 | - | 1.00 | - | 1.00 | - |
| *Late*  *(>13.8 years)* | 0.90  (0.70 – 1.17) | .442 | 0.93  (0.72 – 1.20) | .573 | 0.82  (0.54 – 1.24) | .346 | 0.90  (0.59 – 1.38) | .641 | 0.91  (0.57 – 1.45) | .682 | 0.97  (0.60 – 1.57) | .915 |

Figure S1 Sample size flow diagram

Pregnant women recruited – core ALSPAC sample

(N = 14,541)

Singleton offspring alive at 1 year of age

(n = 13,793)

Sent at least one puberty questionnaire

(n = 6,670)

Female offspring

(n = 6,676)

Complete data on all exposure, outcome, and confounders n = 1,282

Data imputed for all eligible participants

(n = 4,042)

Data on age at menarche from at least one timepoint

(n = 4,042)
